# Obesity Report: Distribution and Pattern by Age and Sex in Heart Failure Cohort over 10 Years in Korea

**DOI:** 10.3390/jcdd12070244

**Published:** 2025-06-26

**Authors:** Joongmin Kim, Geunhee Park, Haeyong Pak, Hyeongsoo Kim, Ji-Yong Jang, Hancheol Lee, Jong-Kwan Park, Seung-Jin Oh, Se-Jung Yoon

**Affiliations:** 1 Division of Cardiology, National Health Insurance Service Ilsan Hospital, Goyang 10444, Republic of Korea; 2 Research Institute National Health Insurance Service Ilsan Hospital, Goyang 10444, Republic of Korea

**Keywords:** obesity, heart failure, prevalence, sex, age

## Abstract

Obesity has been shown to be an independent risk factor for the development of heart failure (HF) and atherosclerotic cardiovascular disease. Here, we tried to analyze the distribution of obesity by age and sex in a 10-year sample cohort of newly diagnosed HF patients in Korea. A total of 35,869 patients newly diagnosed with HF between 2006 and 2015 from a nationally representative random sample of 1,000,000 people were included in this study. The data of age and sex for each subgroup according to body mass index were analyzed and compared with the general population. The obese group accounted for 43.3% of the total, and the frequency of obese patients was the highest among those in their 60s (4561). The proportion of obesity was the highest among those in their 40s (57.7%) and 30s (57.3%) in men and the highest among those in their 60s (52.2%) in women. The underweight group increases with age in both men and women, and the proportion of the high-aged group over 80 years old in the underweight group of women is significantly much higher than that of men. Conclusively, the proportion of obesity is higher than in the general population in HF patients. Obesity patterns analyzed by age were different for each sex.

## 1. Introduction

The recent obesity epidemic has been called a ‘global epidemic’ that encompasses some of the most important health issues facing humans and the environment along with malnutrition and climate change [1,2]. The prevalence of obesity tripled worldwide from 1975 to 2016, according to the World Health Organization (WHO) [3,4].

According to the World Obesity Federation World Obesity Atlas 2022, including the U.S., all countries, including Europe, Africa and the Eastern Mediterranean, are affected by obesity. The number of adults suffering from obesity is expected to increase over the current decade in all countries. When calculated by body mass index (BMI), the highest rates of obesity are found in the Americas for both men and women. Women are more affected by obesity than men in all regions [5]. It is not different in Republic of Korea (hereafter, Korea), where the proportion of adults with a BMI ≥ 25 kg/m^2^ has been increasing rapidly and the prevalence of obesity was 36.3% in 2019 [6,7,8]. Obesity is increasing every year, along with type 2 diabetes, hypertension and dyslipidemia, in Korea [9].

Recent evidence from four European cohorts suggests that individuals with a BMI greater than 35 kg/m^2^ live, on average, 6–7 years less in overall health and 9–10 years less while free from cardiovascular disease, respiratory disease or cancer [10]. The relative risk of developing ischemic stroke, myocardial infarction, type 2 diabetes and cancers in people with obesity or abdominal obesity is higher compared to those without obesity or abdominal obesity. Internationally, data from the Global Burden of Disease (GBD) study show that high-BMI disability is the leading cause of hypertension, ischemic heart disease and stroke [11]. Results from European cohorts with long-term follow-ups of 10 to 20 years suggest that middle-aged or older adults with obesity are at increased risk of ischemic stroke [12,13]. Increased BMI has long been known as a risk factor for heart failure (HF) [14,15,16,17,18] and more recently has also been shown to increase risk for several diseases and mortality [15,19]. As obesity is associated with several metabolic disorders, such as dyslipidemia, inflammation, insulin resistance and endothelial dysfunction [20,21], it has been repeatedly demonstrated to be an independent risk factor for the development of HF and atherosclerotic cardiovascular disease [2,15,16,17,22]. It also alters cardiac structure and ventricular function, as well as central hemodynamics, increasing the risk of arrhythmias and even sudden cardiac death [18,23,24].

HF includes various cardiac dysfunctions resulting from diabetes, hypertension and ischemic heart disease, which can be caused by obesity. Investigating and analyzing the current status of controllable metabolic factors is very important from a preventive standpoint before disease management. Here, we tried to analyze the distribution of obesity by age and sex in a 10-year sample cohort of newly diagnosed HF patients and compared it with the general population.

## 2. Materials and Methods

### 2.1. Source of Data

This study adhered to the tenets of the Declaration of Helsinki, and this study design, using the National Health Insurance Service National Sample Cohort 1.0 database (NHIS-NSC) 2002–2015 project, was reviewed and approved by the Institutional Review Board of the National Health Insurance Service Ilsan Hospital, Gyeonggi-do, Korea (NHIMC 2023-04-002). Written informed consent was waived. Claim data from the Korea National Health Insurance Service (KNHIS) program, the Medical Assistance Program and the Medical Care (Medical Aid) program in Korea, which all citizens are required to subscribe to, are not duplicated or omitted for each person and allow the individual’s medical service behavior to be identified [25,26]. As a result, almost all health system data are centralized in large databases. Patients supported by the KNHIS will be responsible for approximately 20–50% of the total medical expenses when using the healthcare facility, and the medical providers will be required to submit claims of the remaining medical expenses. Claims include data on diagnosis codes, prescriptions, patient information, hospital information, direct medical charges for inpatient and outpatient care, various procedures and dental services. Additionally, the KNHIS uses the Korean Classification of Diseases (KCD), which is a system very similar to the International Classification of Diseases (ICD) [27,28].

This study used the NHIS-NSC 1.0 database 2002–2015 released by the KNHIS in 2018. These data are comprised of a nationally representative random sample of 1 million people, representing approximately 2.1% of the total population of the KNHIS in 2006. These data were generated with a sample representing 48,222,537 Korean residents in 2006 using systematic sampling methods by the KNHIS. The database includes all medical claims submitted from January 2002 to December 2015. This study was a non-interventional, retrospective enrollment study using data of claims from the electronic patient records of the KNHIS for health insurance coverage and bi-annual health screening data.

### 2.2. Population and Study Design

The data used in this study were from a sample cohort of new diagnoses of ‘heart failure’, collected nationwide from 2006 to 2015, targeting approximately 35,867 residents with BMI information over the age of 20. The patient population was based on a first recorded ICD-10 (I50, I09.9, I11, I13, I13.2) diagnosis of HF between 2006 and 2015. The HF group included all patients who underwent inpatient and outpatient treatment for an initial diagnosis of HF (corresponding to KCD codes I50, I099, I110, I130 and I132, as well as International Classification of Diseases, 10th Revision, Clinical Modification [ICD-10-CM] codes I50, I09.9, I11, I13 and I13.2) between 2006 and 2015. Age, sex and anthropometric measures were analyzed.

### 2.3. Definition of Obesity

We used BMI data calculated using anthropometric measures from health check-ups. Obesity was defined as a BMI (weight in kilograms divided by the square of height in meters) ≥25.0 kg/m^2^ in adults, in accordance with the Asia-Pacific criteria of the World Health Organization guidelines and the Korean Society for the Study of obesity guideline. We did not adjust for potential differences in body composition and metabolic risk between Korean and Western populations. In the analyses using BMI, patients were divided into five subgroups: underweight (<18.5 kg/m^2^); normal weight (18.5–22.9 kg/m^2^); overweight (23.0–24.9 kg/m^2^); obese (25.0–29.9 kg/m^2^) and extremely obese (≥30.0 kg/m^2^) [9,29,30,31]. The obesity information of the total population from Statistics Korea was referred for comparison with that of HF patients. As a government agency, Statistics Korea provided annual obesity or BMI data from Korean National Health and Nutrition Examination Survey (KNHANES) data of the Korea Disease Control and Prevention Agency and health checkup statistics of the NHIS, respectively [32,33].

### 2.4. Statistical Analysis

Categorical variables were presented as a percentage of the total group, and the percentage was calculated by dividing it into columns and rows, respectively. The χ^2^ test was used to compare categorical variables, and a significance level of 0.05 was chosen. The analysis was performed using the statistical package SAS System for Windows, version 9.4 (SAS Inc., Cary, NC, USA) in this study.

## 3. Results

### 3.1. BMI and Sex Distribution in Heart Failure

**Figure 1:**
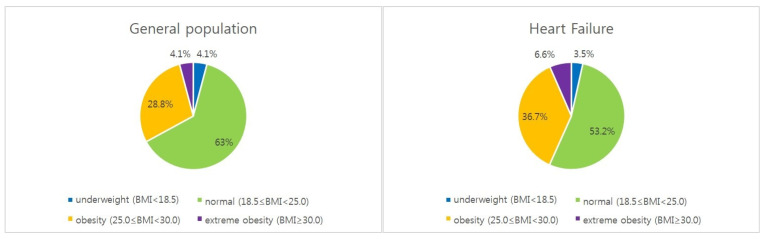
BMI distribution in heart failure (4 subgroups). Distribution of BMI subgroups (4 subgroups) in general health check-up population and heart failure patients. The obese group accounted for 43.3% of the HF population, and it was higher than that of the general health check-up population (32.9%). General health check-up data of National health insurance service from Korean Statistical Information Service (2010−2021).

We analyzed patients diagnosed with HF from a nationally representative random sample of 1 million people over a 10-year period from 2006 to 2015. A total of 35,869 HF patients with anthropometric information including weight and height were analyzed, including 16,876 men and 18,991 women patients. The obesity group of HF accounted for 36.7% of the total and it accounted for 43.3% including group of extreme obesity. It was higher than that of the general health check-up population from 2010 to 2021 (32.9%) (Table 1, Figure 1) [33].

**Figure 2:**
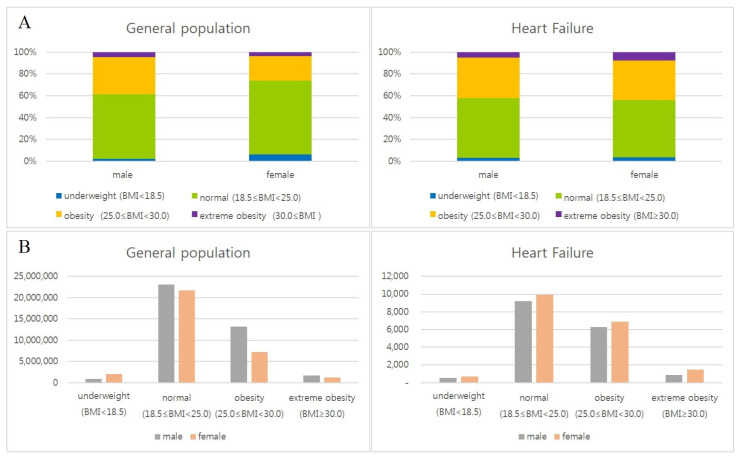
BMI and sex distribution in heart failure. Distribution of BMI subgroups (4 subgroups) by sex in general health check-up population and heart failure patients. The proportion of obese people with a BMI over 25 kg/m^2^ in HF was 42.3 and 44.2% in men and women, respectively, and it was also higher than that of the general health check-up population (38.5 and 26.2% in men and women, respectively) (**A**). The incidence of HF is higher in women across all BMI subgroups compared to the general population (**B**). General health check-up data of National health insurance service from Korean Statistical Information Service (2010–2021).

The proportion of obese people with a BMI over 25 kg/m^2^ in HF was 42.3 and 44.2% in men and women, respectively, and it was also higher than that of the general health check-up population from 2010 to 2021 (38.5 and 26.2% in men and women, respectively) (Table 1, Figure 2A). The incidence of HF is higher in women across all BMI subgroups compared to the general population (Table 1, Figure 2B) [33]. 

### 3.2. Distribution of BMI Subgroups by Age in Heart Failure

The combined frequency of obesity and extreme obesity was the highest among patients in their 60s (4561), followed by those in their 70s (3719) and 50s (3658), respectively. However, in the same age group, the proportion of obesity and extreme obesity was the highest in patients in their 40s (50.4%), followed by those in their 50s (48.4%) and 60s (47.9%), respectively.

**Figure 3:**
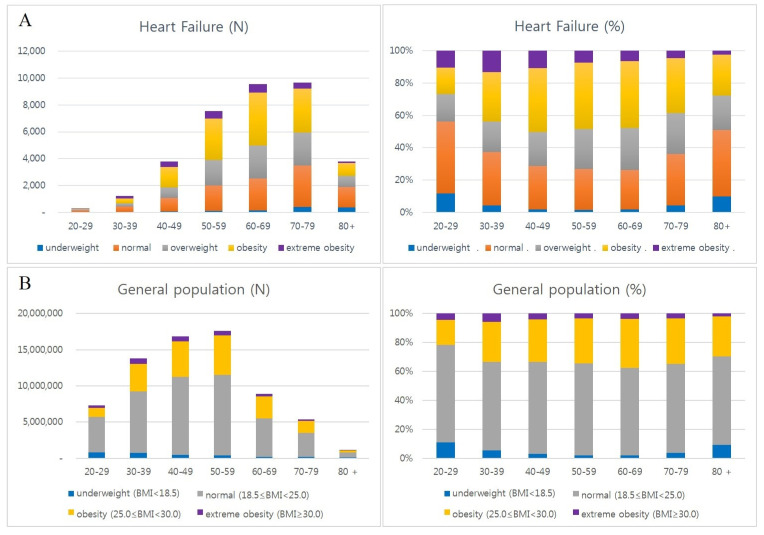
Distribution of BMI subgroups by age in heart failure. Analysis by age in heart failure patients (**A**) and general health check-up population (**B**). The combined frequency of obesity and extreme obesity groups was the highest in those in their 60s (4561), followed by those in their 70s (3719) and 50s (3658), respectively, in the HF group. However, in the same age group, the proportion of obesity and extreme obesity groups was the highest in their 40s (50.4%), followed by those in their 50s (48.4%) and 60s (47.9%), respectively (**A**). Age groups with high proportion of obesity were similar to the health check-up population (40s–60s of HF group vs. 30s–70s of check-up group) (**B**). General health check-up data of national health insurance service from the Korean Statistical Information Service (2010–2021).

The pattern of obesity rate in each age group was similar to that of the entire health screening population (40s–60s of HF group vs. 30s–70s of check-up group) (Table 2, Figure 3) [33].

The proportion of extreme obesity and obesity groups has declined with age in the same age group.

### 3.3. Distribution of BMI Subgroups by Age and Sex in Heart Failure

**Figure 4:**
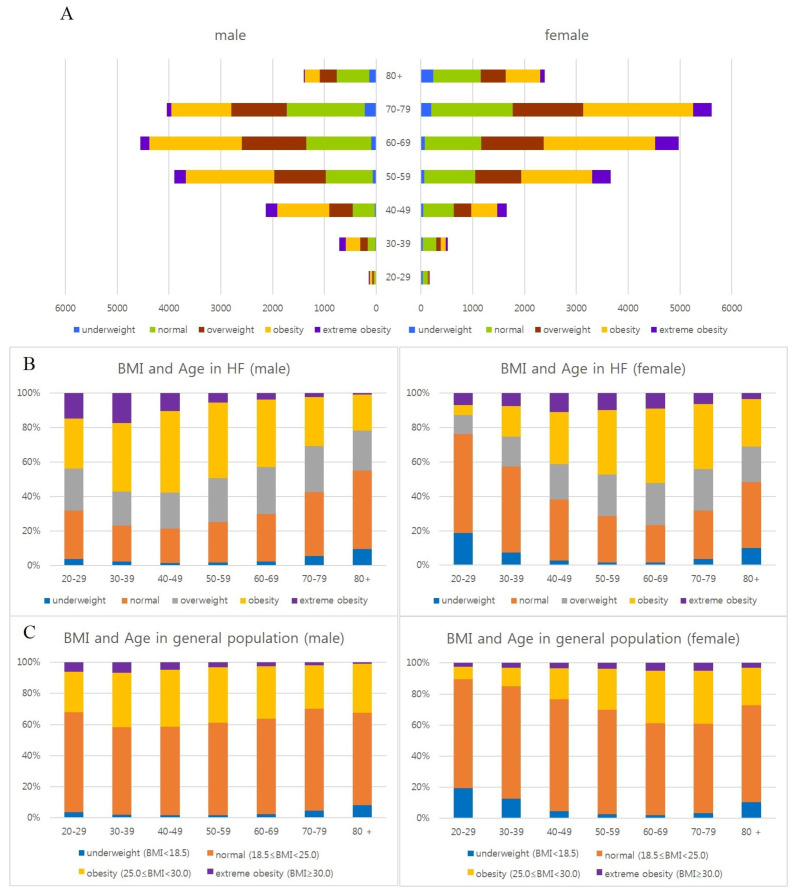
Distribution of BMI subgroups by age and sex in heart failure (2006–2015). Analysis by age and sex in heart failure patients (**A**,**B**) and general health check-up population (**C**). In the same age group, the proportion of obesity was the highest in men in their 40s (57.7%) and 30s (57.3%), and women in their 60s (52.2%) in heart failure. The proportion of the obese group has declined with age in older people, and on the contrary, that of normal and underweight groups has gradually increased in the same age group in both sexes (**B**). The pattern of the proportion of obesity by age and sex was similar to that of the health check-up group of the general population (**C**). General health check-up data of national health insurance service from Korean Statistical Information Service (2010–2021).

In the same age group, the proportion of extreme obesity and obesity was the highest among men in their 40s (57.7%) and 30s (57.3%) and women in their 60s (52.2%). The proportion of extreme obesity and obesity has declined with age in older people, and on the contrary, that of normal and underweight groups has gradually increased in the same age group in both sexes. The pattern of proportion of obesity by age and sex was similar to that of the health check-up group of the general population (Table 3, Figure 4) [33].

In summary, more than half of middle-aged and early elderly people of HF are obese or extremely obese, but it has begun to decrease to less than half in their 60s for men and 70s for women.

**Figure 5:**
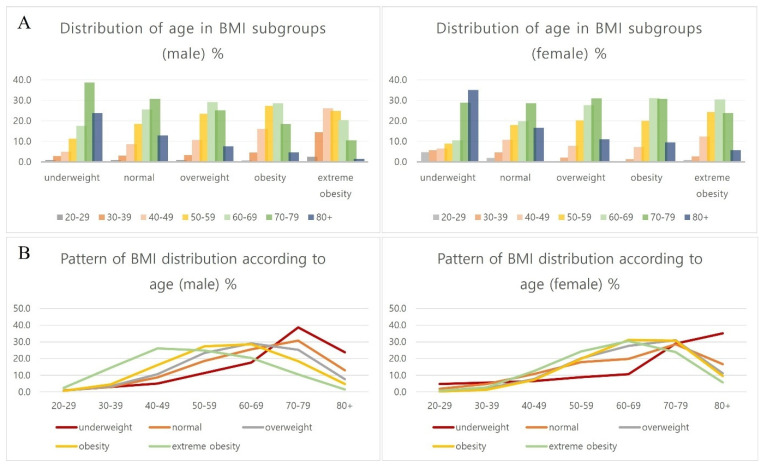
Distribution of age and sex according to BMI subgroups in heart failure. The underweight group increases with age in both men and women, and the proportion of high-aged group (over 80 years old; dark blue bar) in the underweight group of women is much higher than that of men (**A**). The underweight group (red line) is gradually increasing as the patients get older in both men and women (**B**).

The underweight group increases with age in both men and women, and the proportion of the high-aged group over 80 years old in the underweight group of women is much higher than that of men (Table 3, Figure 5).

## 4. Discussion

This study is the first report to analyze the distribution of obesity and differences by sex and age in heart failure patients in Korea. By leveraging a nationally representative dataset, this study strengthens the understanding of how obesity distribution differs by age and sex, offering a comparison to general population trends. This study showed a higher proportion of obesity in HF subjects than in the general population, and men showed the highest proportion of obesity at a much younger age than women. The proportion of obesity was the highest men in their 40s and 30s and the highest in women in their 60s; this phenomenon was similar in the general population. Additionally, the proportion of the obese group has declined with age in extremely older people, and that of normal and underweight groups has gradually increased in both men and women.

By comparing heart failure patients with national health check-up data, this study tried to contextualize obesity prevalence relative to a broader epidemiological backdrop. And this comparison will strengthen public health relevance by suggesting the need for tailored obesity management strategies in heart failure patients.

Obesity is known to be the main cause of many metabolic diseases, and its incidence is increasing worldwide, so it is sometimes called a ‘syndemic’ [1,2]. Obesity is expected to continue to increase worldwide in the future due to lifestyle habits such as reduced physical activity, various non-face-to-face systems that are developing and high-salt and high-calorie eating habits [6]. What is even more unfortunate is that it is expected to occur in younger age groups than before. However, since it is realistically impossible to return to past lifestyles or diets, it is necessary to cultivate a ‘clever population’ through continuous education and promotion to prevent obesity, and it should be also supported by a systematic and simple health check-up system for early diagnosis of comorbidities.

However, investigating the vulnerable group of obesity is of further importance in determining the targets for future health education and examinations, as well as efficiently allocating health finances. Investigating and analyzing the current status of controllable metabolic factors is very important from a preventive standpoint before disease management, and we tried to investigate the proportion of obesity in Korean heart failure patients and analyze the characteristics by sex and age.

The proportion of obesity in HF patients was higher than that of the overall health check-up population in both men and women [33]. This showed a similar pattern compared to the results of a 2015 study using NHIS check-up data (43.3 vs. 32.4 in total, 42.3 vs. 40.7 in men, 44.2 vs. 24.5% in women) [34]. Furthermore, in comparison with the results of the KNHANES data of the Korea Disease Control and Prevention Agency in a similar period to this study (2007–2015), the obesity rate in HF patients is higher than that in the general population (43.3 vs. 32.7 in total, 42.3 vs. 35.1 in men, 44.2 vs. 29.3% in women) [32]. However, considering the older age of heart failure patients than the general population, it would be more accurate to compare the obesity rate in the same age and sex groups rather than mean value in total or each sex.

The interesting point of this study is that, contrary to the initial prediction of the authors, the distribution of the obese group was different for each sex. This study shows that the obesity proportion of both men and women with heart failure did not increase with age and that men have the highest distribution of obesity in their 40s and 30s, while women exhibit this phenomenon in their 60s. Of course, since the BMI we used is a simple number that does not take into account actual body fat, muscle composition ratio or abdominal circumference, it should be considered that middle-aged men’s BMI values have a higher percentage of muscle mass than other ages or women in this study. Considering this, it can also be understood that the proportion of underweight and normal groups increased relatively among extremely older people with reduced actual muscle mass. The decline in BMI in the older group with age may be greatly due to muscle loss and the catabolic phenomenon of HF.

As mentioned in many studies, this reveals the limitations of BMI as an obesity indicator, but at the same time, BMI is widely used as the most objective and simplest surrogate marker because there is no other more reasonable and easier indicator available [35,36,37,38]. However, the complexity of obesity cannot be captured or defined simply by BMI, and BMI does not take into account the location or amount of body fat relative to muscle or the weight of the skeleton, which can vary depending on gender, age and race [39,40]. There are other measures that can more broadly assess the poor health caused by high body fat. However, such assessments require complex and well-equipped health systems to address obesity systematically, and they may not be readily utilized or implemented in many current healthcare systems, especially in low- and middle-income countries [5].

So, currently, BMI is usually used for health statistics or epidemiological studies covering the entire population. In the overall population data, BMI and abdominal circumference increase with age in women, and the patterns of the two indices are generally consistent, while for men, BMI is highest in the 30s and 40s, and waist circumference increases with age, so the obesity distribution determined by these two indices does not match by age [8,9,33]. This also shows a similar pattern in the heart failure group of this study. This is consistent with the statistical results of the 2007–2015 KNHANES data. For men, the average obesity rate was highest during the observation period in their 30s and 40s (41.8 and 41.8%, respectively) and gradually decreased with age, while for women, the average obesity rate was highest in their 60s (43.6%) [32]. This appears to be due to potential confounding factors such as menopause, metabolic shifts or cultural dietary habits [41]. If there is a simple, reproducible and more reliable indicator that accurately checks the proportion of fat in the body, it would be possible to evaluate obesity in real life much more accurately and use it as a precise research tool.

According to the results of this study, the proportion of obesity in heart failure patients was higher than that in the general population, but the patterns of distribution by age and sex were not different from those in the general population group. Comparing a specific group of diseases to the general population can help identify their characteristics and differences more clearly. This study shows that, unlike women, men need special attention and advice such as lifestyle modifications, dietary recommendations or pharmacological approaches from an early age against obesity, a major risk factor for heart failure.

### Study Limitations

As mentioned above, the lack of accuracy and refinedness of BMI as an indicator of obesity that should take into account complex body fat and muscle mass is a major limitation. With additional data such as body composition analysis or waist circumference, better results can be expected.

Another important limitation is that the diagnosis of heart failure was defined based on KCD codes, which may be less accurate than diagnoses obtained from medical charts and could lead to underreporting or misclassification. In addition, echocardiographic data, symptoms and NYHA classification in medical records are not included, which is also a limitation. Accurate diagnostic codes in medical records are crucial for medical insurance claims in Korea; however, underreporting, omissions and misclassifications of medical diagnoses may occur. Also, the lack of an analysis of annual changes in obesity in patients with heart failure is another limitation. However, given the increasing prevalence of heart failure and the proportion of obesity in the entire population, it can be predicted that this will also increase. Much better data could have been obtained by comparing outcomes such as mortality or cardiovascular events for the obese and control groups of the entire heart failure group, but such analysis was not conducted. Lastly, when check-up data are used for BMI analysis, there can be a possibility that subjects who could not be screened due to very old age, other severe diseases or poor transportation area may not be included, which can be another limitation of this analysis.

## 5. Conclusions

The proportion of obesity is higher than in the general population in HF patients. Obesity patterns analyzed by age were different in each sex. However, the trend is similar to the general population. Men had higher rates of obesity at younger ages than women.

## Figures and Tables

**Table 1:** BMI and sex distribution in heart failure.

	Men		Women		Total	
	N (Row %)	Column %	N (Row %)	Column %	N	Column %
**Underweight**	559 (45.0)	3.3	683 (55.0)	3.6	1242	3.5
**Normal**	4920 (47.1)	29.2	5529 (52.9)	29.1	10449	29.1
**Overweight**	4258 (49.2)	25.2	4392 (50.8)	23.1	8650	24.1
**Obesity**	6279 (47.7)	37.2	6892 (52.3)	36.3	13171	36.7
**Extreme obesity**	860 (36.5)	5.1	1495 (63.5)	7.9	2355	6.6
**Total**	16,876 (47.0)	100.0	18,991 (53.0)	100.0	35,869	100

BMI, body mass index (kg/m^2^). (*p*-value < 0.0001). Underweight, BMI < 18.5; normal, 18.5 ≤ BMI < 23; overweight, 23 ≤ BMI < 25; obesity, 25 ≤ BMI < 30; extreme obesity, BMI ≥ 30.

**Table 2:** Distribution of BMI subgroups by age in heart failure.

Total Patients									
	Age	20–29	30–39	40–49	50–59	60–69	70–79	80+	Total
Group									
**Underweight**									
N		37	54	72	123	170	413	372	1241
Column %		11.8	4.4	1.9	1.6	1.8	4.3	9.8	
Row %		3.0	4.4	5.8	9.9	13.7	33.3	30.0	100.0
**Normal**									
N		139	405	1016	1898	2346	3085	1547	10,436
Column %		44.4	33.0	26.8	25.1	24.6	32.0	40.9	
Row %		1.3	3.9	9.7	18.2	22.5	29.6	14.8	100.0
**Overweight**									
N		53	230	791	1879	2451	2433	811	8648
Column %		16.9	18.8	20.9	24.9	25.7	25.2	21.5	
Row %		0.6	2.7	9.1	21.7	28.3	28.1	9.4	100.0
**Obesity**									
N		51	374	1503	3083	3932	3273	952	13,168
Column %		16.3	30.5	39.6	40.8	41.3	33.9	25.2	
Row %		0.4	2.8	11.4	23.4	29.9	24.9	7.2	100.0
**Extreme obesity**									
N		33	163	410	575	629	446	97	2353
Column %		10.5	13.3	10.8	7.6	6.6	4.6	2.6	
Row %		1.4	6.9	17.4	24.4	26.7	19.0	4.1	100.0
								
**Total**		313	1226	3792	7558	9528	9650	3779	35,867
(Row %)		0.9	3.4	10.6	21.1	26.6	26.9	10.5	100

BMI, body mass index (kg/m^2^). (*p*-value < 0.0001). Underweight, BMI < 18.5; normal, 18.5 ≤ BMI < 23; overweight, 23 ≤ BMI < 25; obesity, 25 ≤ BMI < 30; extreme obesity, BMI ≥ 30.

**Table 3:** Distribution of BMI subgroups by age and sex in heart failure.

Men									
	Age	20–29	30–39	40–49	50–59	60–69	70–79	80+	Total
Group									
**Underweight**									
N		5	16	28	63	98	216	133	559
Column %		3.5	2.3	1.3	1.6	2.2	5.3	9.5	
Row %		0.9	2.9	5.0	11.3	17.5	38.6	23.8	100.0
**Normal**									
N		40	147	425	910	1254	1508	632	4916
Column %		28.4	20.7	19.9	23.4	27.5	37.3	45.3	
Row %		0.8	3.0	8.6	18.5	25.5	30.7	12.9	100.0
**Overweight**									
N		34	140	450	995	1241	1074	324	4258
Column %		24.1	19.7	21.1	25.6	27.2	26.6	23.2	
Row %		0.8	3.3	10.6	23.4	29.1	25.2	7.6	100.0
**Obesity**									
N		41	282	1006	1711	1789	1154	293	6276
Column %		29.1	39.8	47.2	44.0	39.3	28.6	21.0	
Row %		0.7	4.5	16.0	27.3	28.5	18.4	4.7	100.0
**Extreme obesity**									
N		21	124	224	213	175	90	12	859
Column %		14.9	17.5	10.5	5.5	3.8	2.2	0.9	
Row %		2.4	14.4	26.1	24.8	20.4	10.5	1.4	100.0
								
**Total**		141	709	2133	3892	4557	4042	1394	16,868

**Women**									
	**Age**	**20–29**	**30–39**	**40–49**	**50–59**	**60–69**	**70–79**	**80+**	**Total**
**year**									
**Underweight**									
N		32	38	44	60	72	197	239	682
Column %		18.6	7.4	2.7	1.6	1.4	3.5	10.0	
Row %		4.7	5.6	6.5	8.8	10.6	28.9	35.0	100.0
**Normal**									
N		99	258	591	988	1092	1577	915	5520
Column %		57.6	49.9	35.6	27.0	22.0	28.1	38.4	
Row %		1.8	4.7	10.7	17.9	19.8	28.6	16.6	100.0
**Overweight**									
N		19	90	341	884	1210	1359	487	4390
Column %		11.0	17.4	20.6	24.1	24.3	24.2	20.4	
Row %		0.4	2.1	7.8	20.1	27.6	31.0	11.1	100.0
**Obesity**									
N		10	92	497	1372	2143	2119	659	6892
Column %		5.8	17.8	30.0	37.4	43.1	37.8	27.6	
Row %		0.1	1.3	7.2	19.9	31.1	30.7	9.6	100.0
**Extreme obesity**									
N		12	39	186	362	454	356	85	1494
Column %		7.0	7.5	11.2	9.9	9.1	6.3	3.6	
Row %		0.8	2.6	12.4	24.2	30.4	23.8	5.7	100.0
								
**Total**		172	517	1659	3666	4971	5608	2385	18,978

BMI, body mass index (kg/m^2^). (*p*-value < 0.0001 in both groups). Underweight, BMI < 18.5; normal, 18.5 ≤ BMI < 23; overweight, 23 ≤ BMI < 25; obesity, 25 ≤ BMI < 30; extreme obesity, BMI ≥ 30.

## Data Availability

Data are contained within the article.
